# A comprehensive proteogenomic study of the human Brucella vaccine strain 104 M

**DOI:** 10.1186/s12864-017-3800-9

**Published:** 2017-05-23

**Authors:** Xiaodong Zai, Qiaoling Yang, Kun Liu, Ruihua Li, Mengying Qian, Taoran Zhao, Yaohui Li, Ying Yin, Dayong Dong, Ling Fu, Shanhu Li, Junjie Xu, Wei Chen

**Affiliations:** Laboratory of Vaccine and Antibody Engineering, Beijing Institute of Biotechnology, Beijing, China

**Keywords:** *Brucella abortus* 104 M, Proteogenomic, Genome reannotation, Virulence factor, Protective antigen, Multichromosomal bacteria

## Abstract

**Background:**

*Brucella spp.* are Gram-negative, facultative intracellular pathogens that cause brucellosis in both humans and animals. The *B. abortus* vaccine strain 104 M is the only vaccine available in China for the prevention of brucellosis in humans. Although the *B. abortus* 104 M genome has been fully sequenced, the current genome annotations are not yet complete. In addition, the main mechanisms underpinning its residual toxicity and vaccine-induced immune protection have yet to be elucidated. Mapping the proteome of *B. abortus* 104 M will help to improve genome annotation quality, thereby facilitating a greater understanding of its biology.

**Results:**

In this study, we utilized a proteogenomic approach that combined subcellular fractionation and peptide fractionation to perform a whole-proteome analysis and genome reannotation of *B. abortus* 104 M using high-resolution mass spectrometry. In total, 1,729 proteins (56.3% of 3,072) including 218 hypothetical proteins were identified using the culture conditions that were employed this study. The annotations of the *B. abortus* 104 M genome were also refined following identification and validation by reverse transcription-PCR. In addition, 14 pivotal virulence factors and 17 known protective antigens known to be involved in residual toxicity and immune protection were confirmed at the protein level following induction by the 104 M vaccine. Moreover, a further insight into the cell biology of multichromosomal bacteria was obtained following the elucidation of differences in protein expression levels between the small and large chromosomes.

**Conclusions:**

The work presented in this report used a proteogenomic approach to perform whole-proteome analysis and genome reannotation in *B. abortus* 104 M; this work helped to improve genome annotation quality. Our analysis of virulence factors, protective antigens and other protein effectors provided the basis for further research to elucidate the mechanisms of residual toxicity and immune protection induced by the 104 M vaccine. Finally, the potential link between replication dynamics, gene function, and protein expression levels in this multichromosomal bacterium was detailed.

**Electronic supplementary material:**

The online version of this article (doi:10.1186/s12864-017-3800-9) contains supplementary material, which is available to authorized users.

## Background

*Brucella spp*. are Gram-negative, intracellular bacterial pathogens that can cause brucellosis in both humans and animals [1]. As one of the most common zoonotic diseases, brucellosis is a significant economic and public health problem worldwide with more than 500,000 new cases reported annually [2]. *B. abortus*, *B. melitensis*, and *B. suis* are the most pathogenic strains in humans and have been identified as potential bio-terrorism agents [3]. At present, vaccination is the most effective approach for preventing and controlling brucellosis [4, 5]. The *B. abortus* 104 M vaccine strain is the only vaccine that has been widely used in China for the control and prevention of human brucellosis since its approval by the Chinese Food and Drug Administration in 1965 [6]. This vaccine strain was isolated from the placenta of a sick cow in a former Soviet republic. The strain exhibits strong immunogenicity with low and stable residual toxicity in experimental animals [7].

Although the *B. abortus* 104 M genome has been fully sequenced (CP009625-CP009626), it was annotated using in silico methods with many unannotated genes and errors. Furthermore, a large number of hypothetical proteins (620 of 3,072) have yet to be annotated, thereby limiting our understanding of the biological processes pertaining to this strain [7]. Proteogenomics is an important tool for integrating protein-level information into the genome annotation process, thereby greatly improving genome annotation quality [8]. Therefore, a proteogenomics study is required to both validate the *B. abortus* 104 M genome annotation and re-annotate mis-annotated novel genes.

The underlying molecular and physiological mechanisms that cause possible residual toxicity and immune protection following 104 M vaccine induction remain to be elucidated. In our preliminary work, we have determined the whole-genome sequence of 104 M and conducted a comparative analysis against its homologous virulent strain A13334 [7]. It revealed highly similar genome structures but a set of genes missing between 104 M and A13334 that related to virulence alteration. This observation suggests that the remaining virulence associated genes in the 104 M genome may lead to the residual toxicity associated with the 104 M vaccine. We also observed a number of protective antigens that can promote both humoral and cellular immunity in the 104 M genome. The occurrence of these antigens is likely to result in the enhancement of host defense mechanisms pertaining to bacterial infections. The identification of these pivotal virulence factors and protective antigens at the protein level is likely to be beneficial in the elucidation of mechanisms that underpin residual toxicity and immune protection following induction by the 104 M vaccine.

To this end, we used a proteogenomics approach that combined subcellular fractionation and peptide fractionation to perform whole-proteome analysis and genome reannotation in *B. abortus* 104 M. Virulence factors, known protective antigens and other protein effectors that are critical for bacterial virulence and vaccine protection were identified at the protein level. Moreover, differences in protein expression levels between the small and large chromosomes in this multichromosomal bacterium were analyzed. This study presents a comprehensive *B. abortus* 104 M proteome analysis while also providing a platform to aid in the understanding of the molecular mechanisms pertaining to the 104 M vaccine.

## Results and discussion

### Proteogenomic strategy for the analysis of *B. abortus* 104 M

This study incorporated a genome-wide protein identification survey of *B. abortus* 104 M using in vitro culture conditions, thereby facilitating refinement of previous genome annotations. Traditional approaches to proteomics analyses of *B. abortus* based on two dimensional gel electrophoresis (2-DE) and high performance liquid chromatography (HPLC) coupled with mass spectrometry (MS) have proved successful; however, hydrophobic and low abundance proteins are under-represented using this approach [9–14]. To enhance coverage of the expressed genome, different strategies for subcellular and peptide fractionation were combined with subsequent LC-tandem mass spectrometry (MS/MS) analysis to resolve *B. abortus* proteins for global analysis [15]. In the present study, we performed subcellular fractionation prior to protein extraction to reduce interference. Whole cell protein and membrane protein extracts were prepared for strain 104 M. Peptide fractionation prior to analysis is an alternative method that achieves higher proteome coverage. Complexity reduction was achieved by SDS-polyacrylamide gel electrophoresis (PAGE) and strong cation exchange (SCX) HPLC separation [16].

**Fig. 1:**
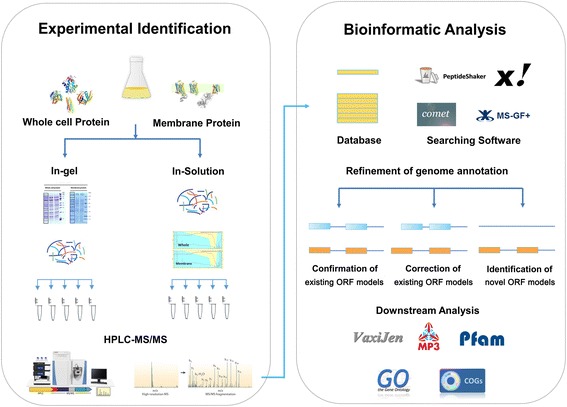
Experimental identification and bioinformatics analysis workflow of the proteogenomics study. Whole cell protein and membrane protein extracts were prepared from *B. abortus* 104 M cultures grown to exponential phase. Complexity reduction was achieved by SDS-PAGE and SCX HPLC separation. After protein extraction and pre-fractionation, enzymatically digested proteins or peptides were analyzed by LC-MS/MS. All spectra were searched against the six-frame (6 F) database and the UniProt database by SearchGUI. Identified peptides were mapped to the 104 M genome and annotation was refined at three levels: (i) confirmation of the existing open reading frame (ORF) models; (ii) refinement of the existing ORF models; and (iii) identification of novel ORF models. Subsequently, the protein physical\chemical property and function were analyzed

In total, 90 samples were generated and subjected to LC-MS/MS analysis using a linear trap quadrupole (LTQ) Obitrap Velos mass spectrometer. The workflow of this study is summarized in Fig. 1. The raw MS data were searched against two databases: (i) a UniProt protein database (UP000064067) and (ii) a 6 F translated genome database of *B. abortus* 104 M (Additional file 1: Table S1) [17]. The search was conducted using SearchGUI and required four different search algorithms (X!Tandem, MS-GF+, Comet and PeptideShaker). Peptides and proteins were inferred from the spectrum identification results using PeptideShaker. Peptide spectrum matches (PSMs), peptides and proteins were validated at a 1.0% false discovery rate (FDR) threshold estimated using the decoy hit distribution (Additional file 1: Table S2-S4). The distribution of precursor mass deviations and the identified peptides that were observed following MS analysis are presented in Additional file 2: Figure S1.

### Analysis of *B. abortus* 104 M protein expression

**Fig. 2:**
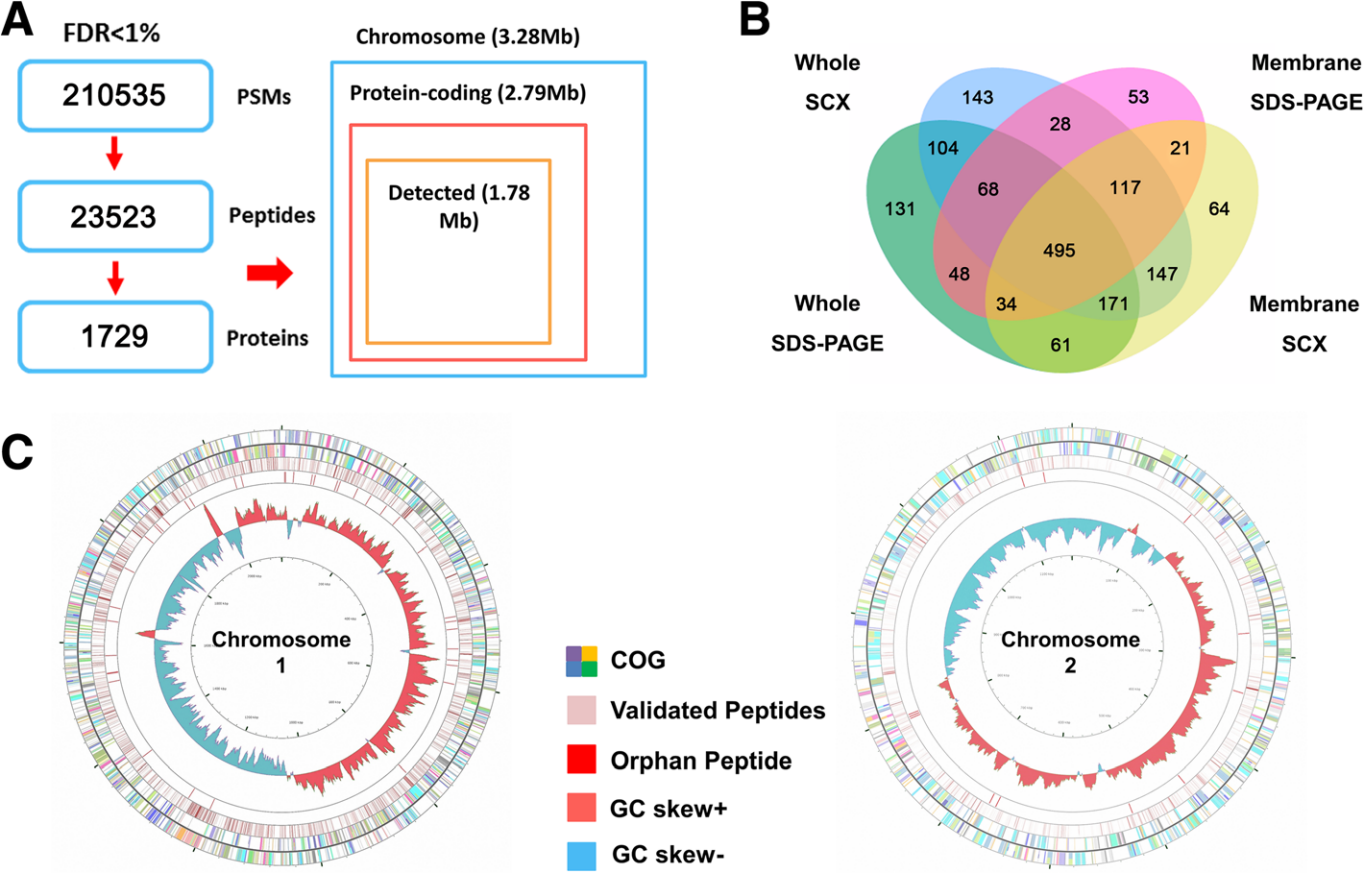
Overview of *B. abortus* 104 M protein expression. **a** Overview of the proteomic evidence for predicted protein-coding genes in the *B. abortus* 104 M genome. **b** Venn diagram of proteins identified by four different fractionation methods: whole SDS-PAGE (whole cell protein with SDS-PAGE fractionation), whole SCX (whole cell protein digestion with SCX fractionation), membrane SDS-PAGE (membrane protein with SDS-PAGE fractionation) and membrane SCX (membrane protein digestion with SCX fractionation). **c** Proteome landscape of *B. abortus* 104 M. Peptides were blasted against the 104 M genome using Circos. The concentric circles from the center to the periphery represent (i) *B. abortus* 104 M chromosomes, (ii) GC content, (iii) orphan peptides identified only in this study, (iv) total peptides identified in this study, and (v) clusters of orthologous groups (COG) annotation of proteins encoded by 104 M genome

We mapped the unique peptides identified in this study to the *B. abortus* 104 M UniProt database (3,072 proteins), and only proteins that were identified by at least two unique peptides were confirmed. A total of 1,729 proteins (1,224 proteins encoded by chromosome 1 and 505 encoded by chromosome 2) were identified, covering approximately 56.3% of the predicted proteome (Fig. 2, Additional file 1: Table S2). Approximately 85.1% (2.79 Mb) of the *Brucella* genome is protein-coding with annotations in the 104 M database, and the expressed genome corresponded to 63.8% (1.78 Mb) of the annotated protein-coding regions. Each identified protein was represented with an average sequence coverage of 34.9% (Additional file 2: Figure S1).

The proteins were identified following in-gel fractionation (SDS-PAGE), in-solution fractionation (SCX), whole cell protein fractionation and membrane protein fractionation (Fig. 2b). Many of the proteins that were uniquely identified in the membrane fractions had relatively extreme grand average of hydropathy (GRAVY) scores (Additional file 2: Figure S2). Large proteins were more likely to be identified after SCX separation than by SDS-PAGE and approximately all unique proteins with molecular weights (MWs) greater than 90 kDa were identified by SCX (Additional file 2: Figure S2). This further emphasizes the advantage of membrane fractionation and SCX for protein separation. Lamontagne et al. identified 621 proteins in preliminary work using three protein separation approaches: SDS-PAGE, isoelectric focusing, and off-line 2-D peptide chromatography [11]. In this study, a combination of subcellular fractionation and peptide fractionation increased the number of membrane and cytoplasmic proteins identifiable by mass spectrometry; This approach provided a significantly higher degree of discrimination and enhanced coverage of the expressed genome.

### Physical and chemical property distributions of identified proteins

**Fig. 3:**
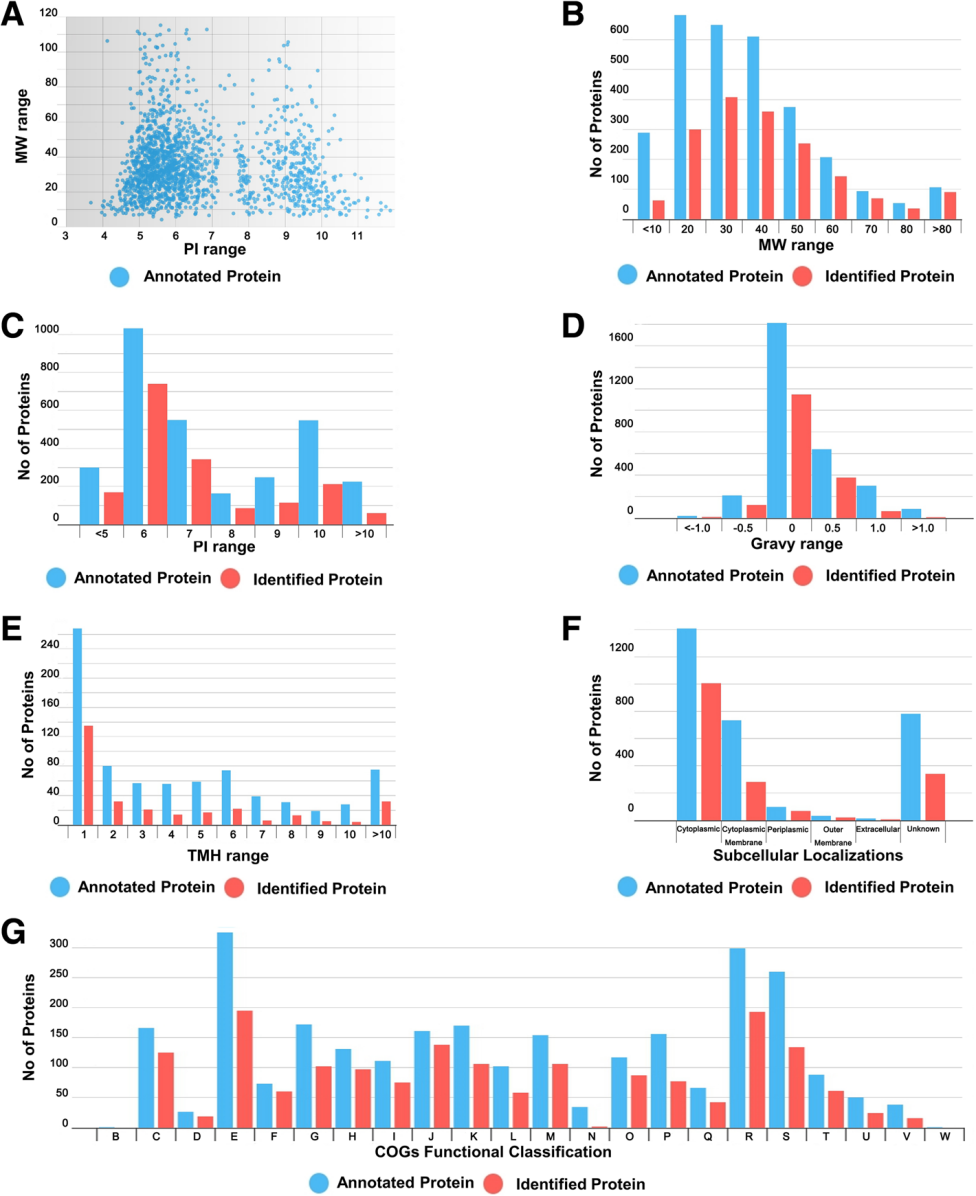
Distribution of the predicted and identified proteins in the *B. abortus* 104 M genome according to their molecular function. **a** Distribution of pI values and MW ranges. Distribution of identified protein **b** MW, **c** pI, **d** GRAVY, **e** TMH, **f** subcellular localization and **g** functional classification of proteins identified according to clusters of orthologous groups (COGs). Annotated and identified proteins are shown in *blue* and *red*, respectively

The proteins identified in this study covered a wide range of MW and isoelectric point (pI) values (Fig. 3a) [18]. The theoretical MW distribution for the identified proteins ranged from 4.7 kDa (Entericidin EcnAB protein) to 320.4 kDa (NdvB protein). Moreover, approximately 85.0% (1,469 of 1,729) of all the identified proteins had MWs in the range 10–60 kDa (Fig. 3b). The pI values of the identified proteins ranged from 3.67 to 11.88. These values are similar to those observed for the total proteome (Fig. 3c). Approximately 67.0% of the proteins identified in the 104 M database had pI values between 6 and 8. Eleven proteins with high pI scores (≥11) and 20 with high MW (≥120 kDa) were beyond the general separation limits of 2-DE.

The GRAVY values for all of the identifications (average −0.012) were used to evaluate protein hydrophilicity and hydrophobicity (Fig. 3d). A total of 58 proteins were identified with high GRAVY scores (≥0.6); such proteins are so hydrophobic that they are rarely detected by 2-DE due to their susceptibility to precipitation during isoelectric focusing. A total of 301 potential membrane proteins with at least one transmembrane helix (TMH) were identified (Fig. 3e) [19]. Among these, the number of predicted TMHs ranged from one to 25. The subcellular localization of identified proteins was predicted using Cello2.5 (Fig. 3f) [20]. Two hundred and eighty-three proteins (16.4%) were predicted to be localized to the cytoplasmic membrane and 1,007 (58.2%) of the proteins were identified as cytoplasmic, which is consistent with findings from previous studies [11]. Each identified protein was classified based on clusters of orthologous groups (COG) and associated with a functional category (Fig. 3g) [21]. Among the 1,729 identified proteins, 1,570 were annotated in the COG database. Many of the identified proteins were involved in amino acid transport and metabolism, translation, ribosomal structure and biogenesis, energy production and energy conversion. All of these categories contain key functional proteins that are important for cell growth and reproduction.

### Identification of virulence factors and function analysis

*Brucella* is unusual because it does not harbor typical virulence factors. Instead, its virulence appears to be an integrated aspect of its physiology, exhibiting a tendency to invade and persist in the human host through inhibition of programmed cell death [22, 23]. As previously stated, the genomic analysis of *B. abortus* 104 M revealed the presence of some virulence associated genes in the 104 M genome which may be related to the vaccine residual toxicity. In this study, we confirmed the presence of 104 M-specific virulence factors of 104 M at the protein level following cell culture. The majority of these factors were observed to be involved in intracellular survival, two-component regulatory systems, and the VirB secretion or transport system (Table 1) [24]. For example, *Brucella* cyclic β-1,2-glucan (CβG) is a key virulence factor that interferes with the maturation of the *Brucella*-containing vacuole and consequently, prevents its fusion with lysosomes [25]. The enzyme responsible for the synthesis of CβG (Cyclic β-1,2-glucan synthase protein, Cgs) was represented by 54 peptides. Similarly, the WbkA and WbkC proteins, which were represented by six and 14 peptides, respectively, are involved in the synthesis of the O-polysaccharide and its translocation to the periplasm [26]. In addition, the BvrR/BvrS sensory-regulatory system, which plays an important role in the stealth program, was represented by 23 and eight peptides, respectively [27]. Moreover, T4SS, which is encoded by the Virb operon (Virb8, represented by three peptides), is also a vitally important factor used for the translocation of virulence factors into mammalian cells and is required for *Brucella* trafficking diversion [28]. Finally, in total 14 pivotal virulence factors in 104 M were identified at the protein level following cell culture. We speculate that the identification of these factors will help us to elucidate the mechanisms that underpin residual toxicity pertaining to the 104 M vaccine.

**Table 1:** The virulence factors and known protective antigens of *B. abortus* 104 M identified in this study

Function Group	Protein Accessions	Name	Description	Protein Length	Identified Peptides
Virulence factors	A0A0M5MHD4	Cgs	Cyclic β-1,2-glucan synthase	2867	135
	A0A0M4TBI0	Gmd	GDP-mannose 4,6-dehydratase	362	46
	A0A0M4SH46	Pgm	Phosphoglucomutase	543	36
	A0A0M3UZI1	BvrR	Transcriptional regulator	239	23
	A0A0M5MBG4	Per	Perosamine synthetase	367	19
	A0A0M3UYN6	Wzt	Teichoic acid ABC transporter ATPase	252	16
	A0A0M4SAL4	WbkC	GDP-mannose 4,6-dehydratase	259	14
	A0A0M4TBJ0	ManAoAg	Mannose-6-phosphate isomerase	390	11
	A0A0M5MBI2	ManCoAg	Mannose-1-phosphate guanyltransferase	474	10
	A0A0M5MCJ0	BvrS	Histidine kinase	601	8
	A0A0M4S2A5	WbkA	Glycosyl transferase family	372	6
	A0A0M5MH13	RicA	Acetyltransferase	175	5
	A0A0M4TDU8	VirB8	Conjugal transfer protein	239	3
	A0A0M4SA70	BtpA	Molecular chaperone	275	2
Protective antigens	A0A0M4SJF6	DnaK	Molecular chaperone DnaK	637	110
	A0A0M4TBF8		Trigger factor	485	77
	A0A0M5MBQ0	CobB	Cobyrinic acid a,c-diamide synthase	436	15
	A0A0M4TD92	GapA	Glyceraldehyde-3-phosphate dehydrogenase	335	81
	A0A0M4S0A3	SurA	Molecular chaperone SurA	318	17
	A0A0M4S1G5	OmpA	Membrane protein	261	13
	A0A0M4SJ84	Bp26	Membrane protein	250	9
	A0A0M4SZK3	Omp25	Membrane protein	213	22
	A0A0M4SCZ0	DnaJ	Molecular chaperone DnaJ	377	20
	A0A0M4S9R8	SodC	Superoxide dismutase	173	47
	A0A0M3UYK8		Invasion protein	173	32
	A0A0M4SDF8	Omp19	Membrane protein	177	13
	A0A0M4TD78	Omp16	Membrane protein	168	17
	A0A0M3V026	Ferritin	Bacterioferritin	161	9
	A0A0M4T393	RibE	Riboflavin synthase subunit beta	158	11
	A0A0M4TCK8	L7/L12	50S ribosomal protein	124	38
	A0A0M4SID4	AsnC	ArsR family transcriptional regulator	159	25

### Identification of known protective antigens and function analysis

The efficacy of the 104 M vaccine against brucellosis has been largely confirmed following its widespread application in China; however, the predominant protective mechanisms associated with the vaccine remain to be elucidated. Interestingly, almost all of the widely reported putative *Brucella* protective antigens listed in the Protegen database were observed to harbor high peptide numbers (Table 1) [29, 30]. For example, 110 peptides derived from the molecular chaperone, DnaK, were identified. This is a member of the highly conserved 70-kDa heat-shock protein (hsp70) family, which assists in folding nascent polypeptide chains and is a known protective antigen [31]. In addition, 37 peptides were identified to be derived from the L7/L12 ribosomal protein, which is a major constituent of the *Brucella* nucleoprotein fraction used in *Brucella* skin tests as a vaccine candidate [32]. Moreover, the outer membrane proteins of *B. abortus*, including the Omp16, Omp19, and Omp25 potential immunogenic antigens, which have been widely explored as subunit vaccines, were represented by 17, 13, and 22 peptides, respectively [33]. Similarly, 54 peptides derived from the antigen periplasmic binding protein P39 were reported. The latter has been reported to be a T-cell immunodominant *Brucella* antigen that induces a Th1-type immune response [34]. Furthermore, peptides derived from other confirmed or putative protective antigens that induce different levels of protective immunity and cellular immune responses against brucellosis, such as trigger factor (TF) chaperone protein, Cu–Zn superoxide dismutase (SOD) proteins, and the transcriptional regulatory proteins AsnC, were also identified in this study [5, 30, 35]. Taken together, our results indicate that *B. abortus* 104 M expresses 17 known protective antigens under in vitro culture conditions. These antigens could promote both humoral and cellular immunity, resulting in the enhancement of host defense mechanisms pertaining to subsequent bacterial infections. Our analysis of protective antigens provides the basis for further research to elucidate the mechanisms of immune protection induced by the 104 M vaccine.

### Identification of hypothetical proteins and function analysis

**Fig. 4:**
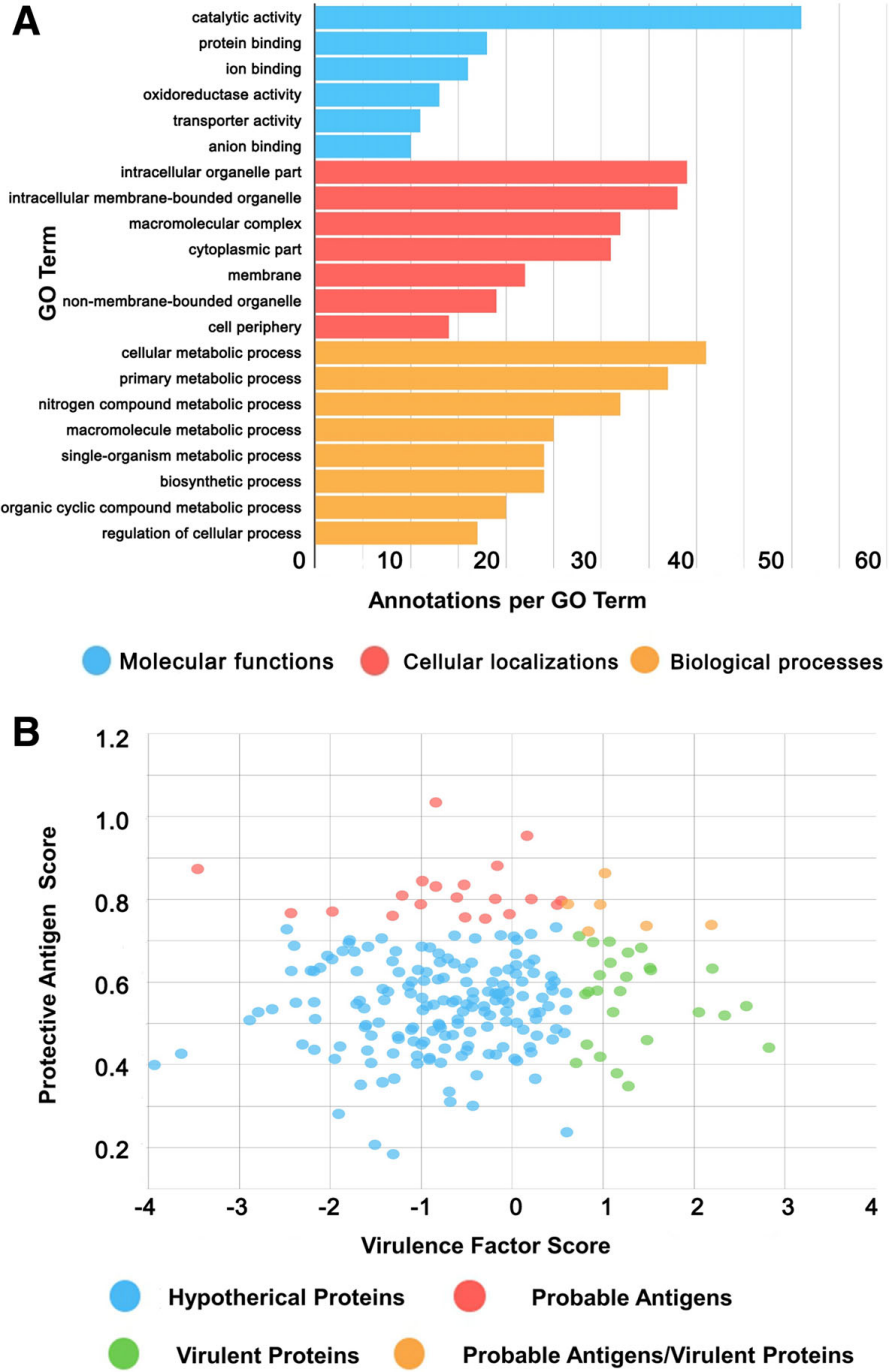
Function analysis of the hypothetical proteins identified in *B. abortus* 104 M. **a** Biological processes, molecular functions, and cellular localizations of all identified hypothetical proteins with GO terms as assigned by Blast2GO. **b** Distribution of the main “Probable Antigens” and “Virulent Proteins” among the identified hypothetical proteins

Since a large number of hypothetical proteins (620 of 3,072) remain to be annotated, our understanding of the biological processes associated with this strain is limited. In this study, 218 hypothetical proteins were identified in the proteomics analysis of 104 M (Additional file 1: Table S5). Among these, 69 hypothetical proteins were allocated gene ontology (GO) terms as assigned by Blast2GO (Fig. 4a) [36]. Many of the identified hypothetical proteins were involved in cellular metabolic process with catalytic activity. In addition, 25 of the hypothetical proteins (with scores higher than the threshold of 0.7) were identified as “probable antigens” following alignment-independent prediction by the VaxiJen server [37]. Thirty-two of the hypothetical proteins (with scores higher than the threshold of 0.6) were identified as “virulent proteins” by the MP3 tool [38]. Distribution of the main “probable antigens” and “virulent proteins” among the hypothetical proteins are shown in Fig. 4b. Twenty-one unique peptides were mapped to a hypothetical protein (A0A0M4S069) re-annotated as “Antifreeze protein” in 104 M. The “Antifreeze protein” has both a high VaxiJen score and MP3 score and is likely be involved in antigenicity and virulence (Additional file 1: Table S5). In summary, 218 hypothetical proteins of *B. abortus* 104 M that were functionally uncharacterized in preliminary studies were confirmed at the protein level during this study. In addition, several of these hypothetical proteins were predicted to be “probable antigens” or “virulent proteins”, which may have some influences on the bacterial virulence and vaccine protection. The conservation of the new virulence factors and protective antigens were assessed at both the nucleotide and predicted amino acid levels using the genome sequences of 18 different *B. abortus* strains [39]. Overall, these analyses demonstrate that thus the newly identified virulence factors, and protective antigens were well conserved among the analyzed isolates.

### Refinement of genome annotation by proteogenomic analysis

All spectra were searched against the 6 F database. The detected peptides were further analyzed via the Visual Exploration and Statistics to Promote Annotation (VESPA) tool and were then mapped to the *B. abortus* 104 M genome [40]. Of these, 74 peptides were orphans that did not map to any annotation in the 104 M genome (Additional file 1: Table S6). Based on our analysis, we identified six novel ORFs and modified three existing ORF models (Table 2). All of the corrections and identifications were assigned to at least two unique orphan peptides. Blast analysis of these novel annotation ORFs across related species revealed the presence of orthologous for all of the novel unannotated ORFs; the analysis also revealed errors in other *B. abortus* strains [41].

**Table 2:** The novel ORFs and annotation errors of *B. abortus* 104 M identified in this study

Proteogenomic Analysis Group	Six-frame Accessions	Description	Protein Length	Identified Peptides
Novel ORFs	NZ_CP009625_614	30S ribosomal protein S1	582	24
	NZ_CP009625_3701	Aldehyde dehydrogenase	301	3
	NZ_CP009625_52863	Transporter	287	2
	NZ_CP009625_60998	Hypothetical protein	157	41
	NZ_CP009625_73594	Phosphoglucomutase	568	6
	NZ_CP009626_31772	Glycerol-3-phosphate dehydrogenase	218	2
Annotation errors	NZ_CP009625_16344	Peptide chain release factor 2	356	12
	NZ_CP009626_15797	Cytoplasmic protein	247	16
	NZ_CP009626_26949	Virulence protein	479	14

**Fig. 5:**
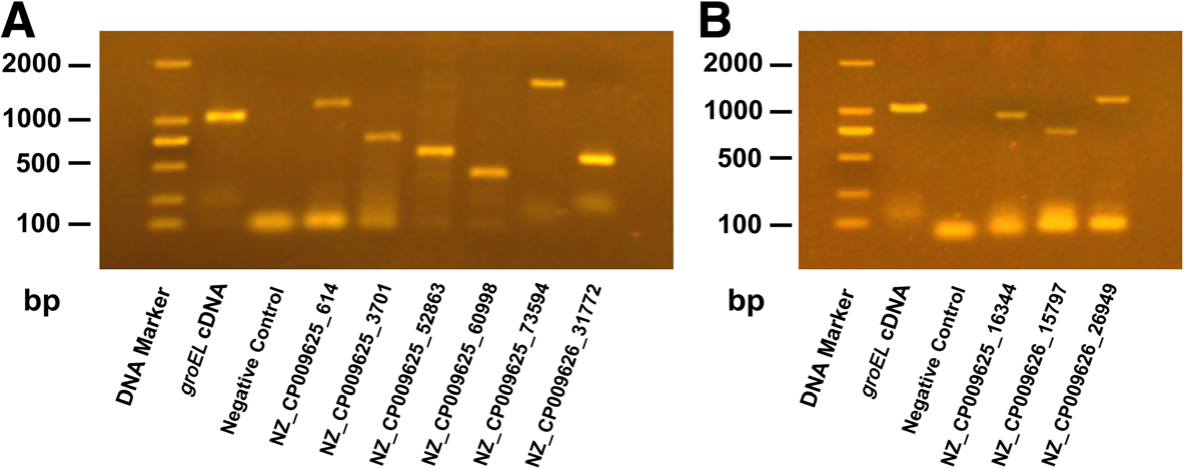
Refinement of *B. abortus* 104 M genome annotation validated by RT-PCR. **a** Novel ORF models and **b** annotation errors of *B. abortus* 104 M were detected and validated at the transcriptional level by RT-PCR. For the PCR amplification: template, novel ORF models and errors cDNA; negative control, RNAs; positive control, *groEL* cDNA

Among the novel ORFs identified, two unique peptides were mapped to a novel ORF (NZ_CP009625_3701) located in the intergenic region between two annotated ORFs (Additional file 2: Figure S3A). This novel ORF contains an Aldedh PFAM structural domain that shares high homology with the aldehyde dehydrogenase protein (A0A0M1WD82) in *B. abortus* [42]. Reverse transcription-PCR (RT-PCR) analysis confirmed that this progenitor ORF is transcribed; suggesting that our identification of novel ORF models was reliable (Fig. 5a). We also identified some erroneously assigned ORF translation initiation sites (TIS). For example, four peptides were first observed to match the upstream region of an existing ORF (NZ_CP009626_26949) that has previously been shown to contain a VirJ PFAM structural domain (Additional file 2: Figure S3B). We found an additional 32 amino acids upstream of the protein start site compared with the annotated genome. Validation of these upstream regions by RT-PCR allowed us to annotate a new accurate start site (Fig. 5b). We analyzed protein TIS by probing N-terminal acetylation modification. The modification directly marked the TIS of protein-coding genes [43]. Based on peptides identified at 1% FDR, and their upstream codons, we confirmed the annotated TIS for 29 genes. List of N-terminal acetylation identified in this study are presented in Additional file 1: Table S7.

In this study, we integrated the *B. abortus* 104 M proteomics information with genome annotation data to identify novel ORFs and validate existing modified ORF models. These observations showed that the identified protein coding genes of the *B. abortus* 104 M genome are well annotated with only six novel ORFs and three existing ORF model errors. Exploring the biological functions of these novel ORFs are under investigation. The refinement of *B. abortus* 104 M genome annotation in this study improved its genome annotation quality. We also confirmed the annotated TIS for 29 genes through N-terminal acetylation analysis. Further research pertaining to N-terminal peptides from other internal digested peptides is required to identify N-terminally modified peptides and gain more information about TISs in *B. abortus*.

### Differential protein expression between the two bacterial chromosomes

*B. abortus* is a multichromosome bacterium, with the associated genome harboring two replicons of unequal sizes [44]. In *B. abortus*, the large chromosome (Chr I, 2.1 Mb) encodes for most of the house-keeping functions, while the small chromosome (Chr II, 1.2 Mb) contains genes mostly of unknown origin and function [45]. The protein expression from multipartite genomes has been poorly investigated. Protein expression and subsequent stability requires a series of linked processes including transcription, processing and degradation. Protein abundance reflects the dynamic balance of these processes and, therefore, may has a positive correlation with expression levels [46].

**Fig. 6:**
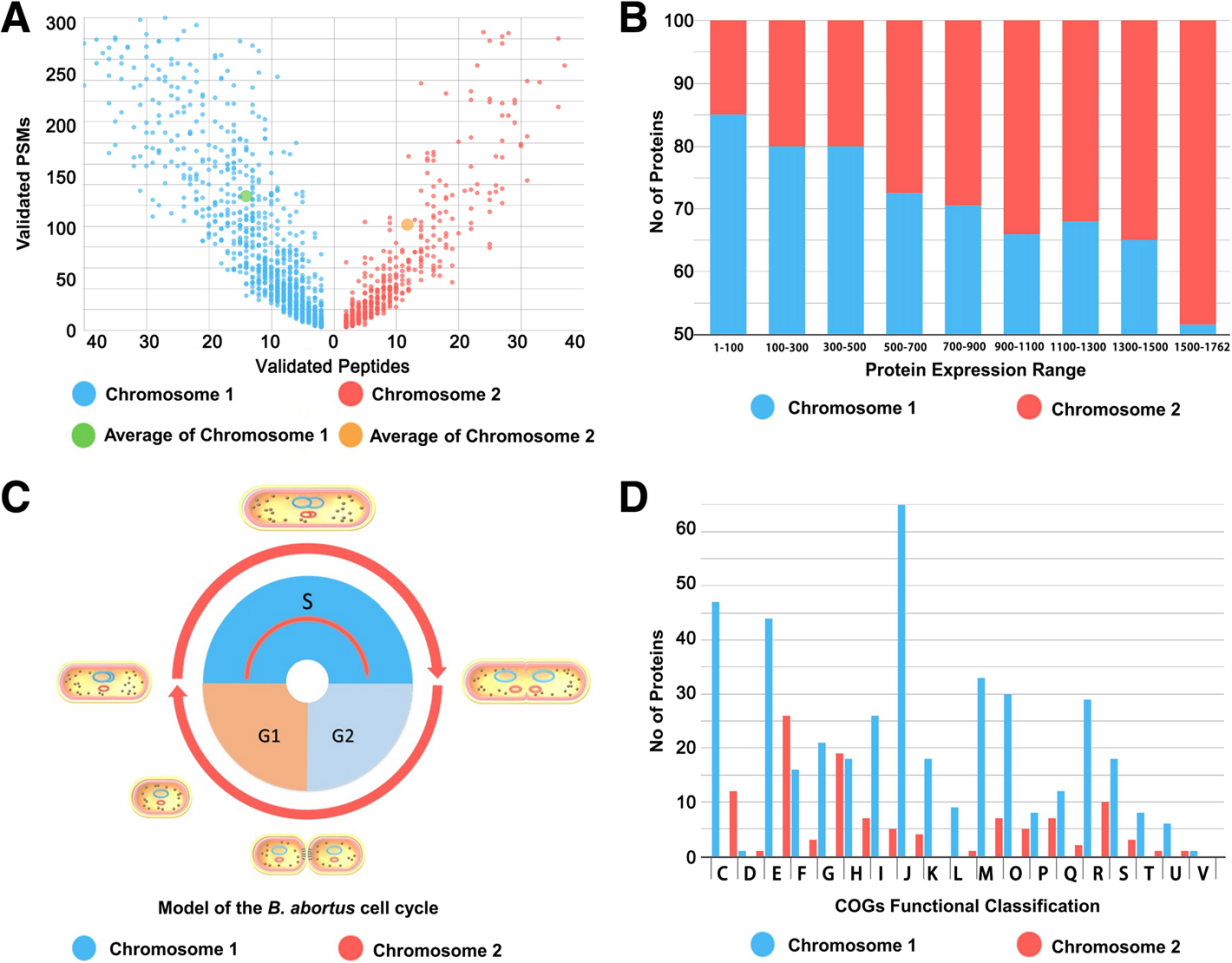
Large Chromosome and Small Chromosome of *B. abortus* 104 M exhibit differences in protein expression. **a** Comparison of protein abundance (peptide numbers and PSM numbers) between the small and large chromosomes of *B. abortus* 104 M. **b** Range of protein abundance expressed by the small and large chromosomes listed in NSAF order (highest level at the top of the list). **c** Model of the *B. abortus* cell cycle. Replication of Chr I is initiated before Chr II. **d** Distribution of COGs within two different chromosomes of the *B. abortus* 104 M genome

In general, an increase in protein abundance typically results in an increase in the number of proteolytic peptides, and vice versa. A resultant increase in the number of (tryptic) digests will usually result in an increase in protein sequence coverage, identified unique peptides, and identified total MS/MS spectra (spectral count) for each protein [47]. The protein abundance associated with 1,729 proteins relative to their chromosomal location in *B. abortus* 104 M were analyzed using identified unique peptides and PSMs of each protein. The Wilcoxon-Rank-Sum test was applied to determine statistical significance. According to our results, the average identified peptide number and PSM number was higher for Chr I compared with Chr II (peptide number 14.03 > 11.81, Wilcoxon-Rank-Sum test *p*-value < 0.01; PSM number 128.9 > 101.6, Wilcoxon-Rank-Sum test *p*-value < 0.01, Fig. 6a).

Since larger proteins would be expected to generate more peptides and, therefore, more spectral counts than smaller proteins, we used normalized spectral abundance factors (NSAF) for comparison of relative quantitation [48]. The NSAF values for each detected protein are presented in Additional file 1: Table S2. After comparing protein abundance between the two chromosomes using the NSAF values, we observed that the relative abundance of proteins pertaining to Chr I was generally higher compared with Chr II (6.364 × 10^−4^ > 4.38 × 10^−4^, Wilcoxon-Rank-Sum test *p*-value < 0.01). The differential protein abundance between the two chromosomes in *B. abortus* 104 M was considered to be statistically significant, which may reflect the differential protein expression between the two bacterial chromosomes.

Proteins were ranked from highest to lowest according to the NSAF values (Fig. 6b). If the expression levels of proteins located on the large and small chromosomes are randomly distributed, then the expected ratio of Chr II proteins would be 1,071/3,072 (or 34.9%) in any arbitrarily selected range. However, according to our results, the actual proportions of Chr II proteins (505/1729, 29.2%) were lower than expected for the upper range of expression levels, except for those proteins with the highest expression levels. For example, of the proteins with expression levels ranging from 1 to 500, only 95 proteins (19%) were located on Chr II. The protein expression levels pertaining to Chr I were generally higher compared with Chr II.

It is well known that the replication of the *B. abortus* genome occurs concomitantly with cell growth and division. The origin and terminator regions of multichromosomes in *B. abortus* have distinct dynamics and localization patterns, whereby the large chromosome is replicated prior to the small chromosome (Fig. 6c) [45]. In addition, the replication of chromosomes results in an increase in the copy number of genes located near origins of replication compared with genes located near termini. This may be a potential reason for the differential protein expression between the large and small chromosome. Moreover, an analysis conducted to elucidate the COG categories of the 500 most highly expressed genes in *B. abortus* 104 M suggests that the proteins associated with rapid cell growth are expressed at relatively high levels. The genes encoding for these proteins were over-represented on the large chromosome and concurrently under-represented on the small chromosome (Fig. 6d).

We also analyzed the GC composition and codon usage between the two chromosomes in *B. abortus* that may affect translation or protein expression. The results showed that there was no significant difference in GC-content (57.2%/57.3%) and codon usage between the two chromosomes (date shown in Additional file 1: Table S8) [7]. The potential reason for differential protein expression may be related to the replication dynamics and gene function differences of the two chromosomes in this multichromosomal bacterium. This study is the first to report differences in protein expression levels in two chromosomes in this multichromosomal bacterium and is in accordance with a similar pattern reported for *Vibrio cholera* following transcriptome analysis [49, 50].

## Conclusions

Brucellosis is a significant economic and public health problem worldwide. The *B. abortus* 104 M vaccine strain has played an important role in the prevention and control of brucellosis in China. In this study, we used a proteogenomics approach that combined subcellular fractionation and peptide fractionation to perform whole-proteome analysis and genome reannotation in *B. abortus* 104 M. In total, 1,729 proteins (56.3% of 3072), including 218 hypothetical proteins, were identified under the culture conditions given in this study. As part of this analysis, 14 pivotal virulence factors, 17 known protective antigens and other important protein effectors that are related to bacterial virulence and vaccine protection were confirmed. These data represents a dataset of proteins identified following growth of 104 M under conventional culture conditions. It is possible that some coding sequences did not express under the conditions chosen in this study. Analysis of differential proteome expression patterns of *B. abortus* in other culture conditions can serve as a starting point in the discovery of protein determinants associated with pathogen adaptation and pathogenesis. These differentially expressed genes potentially promote pathogen adaptation to more complex in vivo environments and, therefore, require further study.

In this study, we also validated the *B. abortus* 104 M genome annotation and re-annotated mis-annotated ORFs; validation improved the quality of the genome annotation. Six novel ORFs and three modified existing ORFs were also identified as part of this study. We confirmed the annotated TIS for 29 genes through N-terminal acetylation analysis. Furthermore, after comparing protein abundance using NSAF values, we observed differential protein expression between the two bacterial chromosomes in in *B. abortus* 104 M. The potential reason for this differential expression may be related to differences in the replication dynamics and gene function pertaining to the two chromosomes; further work is required to verify this hypothesis. In conclusion, this study represents a comprehensive proteogenomics analysis of *B. abortus* 104 M and is likely to improve our understanding of the mechanisms that underpin residual toxicity and vaccine-induced immune protection.

## Methods

### Cell culture and protein extraction

The 104 M strain was grown in tryptic soy broth (TSB) and harvested in the early exponential phase. Whole cell protein samples were prepared as described previously with some modifications [11]. Briefly, cells harvested by centrifugation (7,000 × *g*, 15 min at 4 °C) were washed followed by resuspension in lysis buffer and disruption by ultra-sonication (25% amplitude, 15 min at 0 °C). The resultant suspension was centrifuged (40,000 × *g*, 30 min at 18 °C) and the protein concentrations in the collected supernatants were measured using a BCA protein assay kit (Thermo Fischer Scientific, Waltham, USA).

Total membrane protein samples were also prepared using Triton X-114 phase-separation as described previously [51]. In brief, harvested cells were lysed by bead beating and then centrifuged (7,000 × *g*, 15 min at 4 °C) to remove insoluble material. Triton X-114 (2% v/v concentration) was added to the supernatant and then stirred (10 min at 4 °C) to obtain the protein extract in a single phase. This mixture was then centrifuged (15,000 × *g*, 10 min at 4 °C) to remove residual insoluble matter and incubated at 37 °C for 10 min. Subsequently, the solution was separated into upper (aqueous) and lower (detergent) phases. The detergent phase was collected and proteins were precipitated by acetone. Protein samples were stored at −80 °C for further analysis.

### Protein pre-fractionation and in-gel proteolytic digestion

Whole cell protein and membrane protein extracts were resolved by 12% SDS polyacrylamide gel electrophoresis, stained with Coomassie blue R250 and excised into gel slices, which were then subjected to an in-gel tryptic digestion protocol [51]. In brief, protein-containing lanes were excised from the gels based on molecular weight and local protein amount. Each band was placed into an Eppendorf tube and destained with 30% acetonitrile and 35 mM ammonium bicarbonate until colorless. Gel pieces were treated with pure acetonitrile and dried under vacuum. Sufficient ammonium bicarbonate (at a final concentration of 50 mM) was added to each tube to cover the gel pieces. Trypsin was subsequently added and the tubes were placed on ice for 40 min. All tubes were then incubated at 37 °C for 12 h and centrifuged (12,000 × *g*, 1 min). Peptides were extracted from each supernatant as follows: 50 μl extraction buffer (5% formic acid, 50% acetonitrile) was added and the resultant mixture was centrifuged (12,000 × g, 1 min). Following incubation for 4 min at room temperature, all supernatants were collected into new tubes. Acetonitrile (100%) was added to each tube and these mixtures were mixed by vortexing for 20 min. Following centrifugation (12,000 × g, 1 min), the resultant mixtures were incubated for 4 min at room temperature; the supernatants were subsequently collected. The resultant peptides were dried under vacuum and stored at −80 °C until further use.

### In-solution proteolytic digestion and peptide pre-fractionation

Whole cell protein and membrane protein extracts were reduced (20 min at 25 °C) in 1 mM dithiothreitol and then alkylated in 5.5 mM iodoacetamide (15 min at 25 °C in the dark) [52]. Trypsin was added (1:50, w/w) and proteins were digested in solution overnight before samples were separated by SCX performed on a Dionex UltiMate 3000 (Thermo Fischer Scientific). Tryptic peptides were loaded onto a PolySULFOETHYL A (PolyLC, Columbia, MD, USA) column and eluted with a linear gradient using ACN/potassium phosphate buffers. After the elution, flow-through fractions were collected into fresh Eppendorf tubes. Adjacent fractions were desalted by C18 reversed-phase spin columns, separated into aliquots containing 150 μg peptides, dried under vacuum and stored at −80 °C until further use.

### LC-MS/MS analysis

The nanoAcquity Ultra Performance LC system (Waters Corporation, Milford, USA) was used for peptide separation equipped with a C18 reversed-phase microcapillary trapping (Maisch, Ammerbuch-Entringen, Germany) as described previously [53]. A total of 90 samples (30 samples conducted in triplicate) were loaded and eluted for 40 min using a 5–40% CAN fraction-optimized nonlinear gradient in 0.1% formic acid. Eluted peptides were analyzed using a LTQ Obitrap Velos mass spectrometer (Thermo Fisher Scientific) as previously described [51]. The eluted peptides were electrosprayed with a distally applied spray voltage of 2.0 kV. The mass spectrometry analysis was carried out in a data-dependent manner. Survey scans were performed at a resolution of 30,000 at target values of 1,000,000 ions in the Orbitrap analyzer with maximum allowed fill times of 150 ms over a mass range of 300–1,600 m/z. Finally, the 20 most intense precursor ions were chosen for MS/MS fragmentation by collision-induced fragmentation. The normalized collision energy for the MS/MS was set to 30%, and the transfer tube temperature was maintained at 220 °C. Exclusion of precursor ion masses over a time window of 30 s was used to suppress repeated fragmentation of peaks.

### Database construction and data processing

The protein database used for MS/MS searches was constructed from the UniProt entries for *B. abortus* 104 M and comprised of 3,072 proteins. A six reading frame (6 F) database was constructed based on all six possible ORFs in the 104 M genome using the “getorf” program in EMBOSS, with all sequences between two stop codons regarded as a protein in this database [54]. The 6 F database comprised 115,420 candidate coding sequences for all of the possible proteins (longer than 20 amino acids) encoded by the genome. Peak lists obtained from MS/MS spectra were identified using X! Tandem, MS-GF+, Comet and PeptideShaker. The identification was conducted using SearchGUI [55–57]. The decoy sequences were created by reversing the 104 M UniProt database. The Enzyme specificity was set to trypsin and a maximum of two missed cleavages were allowed. The initial maximal was set to 20.0 ppm as MS1 and 0.5 Da as MS2 tolerances. The fixed modifications carbamidomethylation of C, variable modifications oxidation of M and acetylation of protein N-term were used. Protein identification was conducted against a concatenated target-decoy version of the *B. abortus* 104 M complement of the UniProt database. PeptideShaker provides statistical confidence estimates for each peptide and protein, taking into account protein inference issues. PSMs, peptides, and proteins were validated at a 1.0% FDR using the decoy hit distribution. All of the raw mass spectra files along with the identification results have been deposited into the ProteomeXchange Consortium via the PRIDE partner repository with the dataset identifier PXD005403 and are now available [58, 59].

### Bioinformatics tools for the prediction of proteins

The analysis of unique proteins identified by different proteomics strategies was conducted using Patternlab4.0 [60]. The venn diagram and circular map of the genome were drawn using jvenn and Circos [61, 62]. The theoretical molecular mass and pI, and GRAVY values were predicted using the ExPASy-ProtParam tool [18]. Protein transmembrane helices were predicted using TMHMM 2.0 [19]. The subcellular localization was predicted using the Cello2.5 program [20]. The COGs classification system was employed to examine the distribution of different gene categories [21]. The protective antigens GO analyses were performed using the Blast2Go online tool [36]. The protective antigens probability scores were predicted using the VaxiJen2.0 server [37]. Antigens were specified as pathogenic or nonpathogenic by the MP3 server [38]. The conservation of new virulence factors and, protective antigens were assessed at both nucleotide and predicted amino acid levels between 18 different *B. abortus* strains, using ClustalOmega [63]. The pfam protein family database was used to generate a complete and accurate classification of protein families and domains [42]. All statistical analyses were performed using GraphPad Prism (GraphPad Software, La Jolla, CA, USA).

### Proteogenomic workflow and bioinformatics analysis

All spectra were searched against the 6 F database [16]. The detected peptides (1% FDR) and the *B. abortus* 104 M genome with associated annotations were imported into the VESPA proteogenomic program [39]. Peptides were mapped to the 104 M genome sequence and a set of unique peptides that did not match the annotated proteins of *B. abortus* 104 M (orphan peptides) were identified and then mapped to the unique locations on the 104 M genome. After the analysis, the annotations of existing ORF models were modified and novel ORFs were identified. All annotation identifications and corrections were assigned on the basis of at least two unique orphan peptides.

### RT-PCR validation

The novel ORF models and errors identified in this study were validated at the transcriptional level by RT-PCR as previously described [64]. The total RNA was extracted using the RNeasy Mini Kit (Qiagen, Valencia, CA) and then RNase-free DNase was added to remove any contaminating genomic DNA. The cDNA synthesis was performed using the QuantiTect Reverse Transcription Kit (Qiagen). For the PCR reaction, the novel ORF models and errors cDNA was used as a template, while RNA and *groEL* cDNA were used as negative and positive controls, respectively. The gene-specific primers in this study are listed in Additional file 1: Table S9.

## Additional files

Additional file 1: This file contains supplementary Tables S1-S9. **Table S1.** The 6 F translated genome database of *B. abortus* 104 M used in this study. **Table S2.** List of proteins identified in this study. **Table S3.** List of peptides identified in this study. **Table S4.** List of PSMs identified in this study. **Table S5.** List of hypothetical proteins identified in this study and function analysis. **Table S6.** List of orphan peptides identified in this study. **Table S7.** List of N-terminal acetylation identified in this study. **Table S8.** The codon usage of two chromosomes in *B. abortus* 104 M. **Table S9.** List of primers used for RT-PCR amplifications in this study. (XLSX 38564 kb)

Additional file 2: This file contains supplementary Figures S1-S3. **Figure S1.** Summary of proteome analysis in this study. **Figure S2.** The unique proteins identified by different proteomics strategies in this study. **Figure S3.** Refinement of genome annotation by proteogenomic analysis in this study. (DOC 1842 kb)

## Abbreviations

2-DE: Two dimensional gel electrophoresis
COG: Clusters of orthologous groups
FDR: False discovery rate
GO: Gene ontology
GRAVY: Grand average of hydropathy
HPLC: High performance liquid chromatography
LTQ: Linear trap quadrupole
MS: Mass spectrometry
MS/MS: Tandem mass spectrometry
MW: Molecular weights
NSAF: normalized spectral abundance factors
ORF: Open reading frame
PAGE: Polyacrylamide gel electrophoresis
pI: Isoelectric point
PSMs: Peptide spectrum matches
RT-PCR: Reverse transcription-PCR
SCX: Strong cation exchange
TIS: Translation initiation site
TMH: Transmembrane helix
TSB: Tryptic soy broth
