# Supplementary material for: A comprehensive proteogenomic study of the human Brucella vaccine strain 104 M
Source: BMC Genomics. 2017 May 23;18:402. doi: 10.1186/s12864-017-3800-9 (PMC5442703; doi:10.1186/s12864-017-3800-9)
Supplement: Supplementary file 2 — This file contains supplementary Figures S1-S3. Figure S1. Summary of proteome analysis in this study. Figure S2. The unique proteins identified by different proteomics strategies in this study. Figure S3. Refinement of genome annotation by proteogenomic analysis in this study. (DOC 1842 kb) [file 12864_2017_3800_MOESM2_ESM.doc]

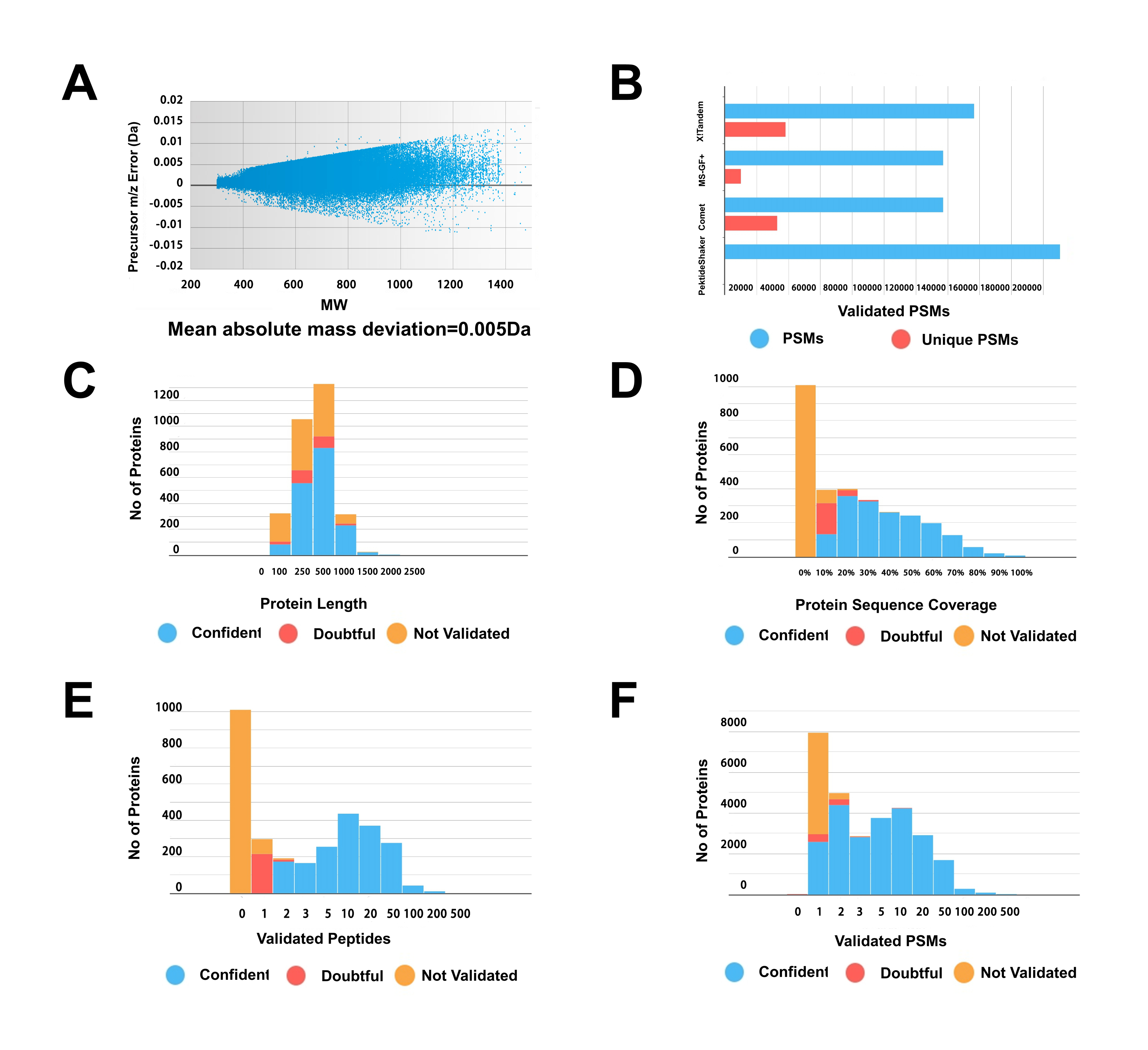


**Figure S1 Summary of proteome analysis in this study.** (A) Distribution of precursor mass deviations and achieved coverage of *B. abortus* 104M genome from mass spectrometry (MS) data. (B) Identification of peptides following X! Tandem, MS-GF+, Comet and PektideShaker searches. (C) Distribution of identified protein length. (D) Distribution of sequence coverage of identified proteins. (E–F) Distribution of the number of identified peptides observed for all identified genes, and the number of validated peptide spectrum matches (PSMs).


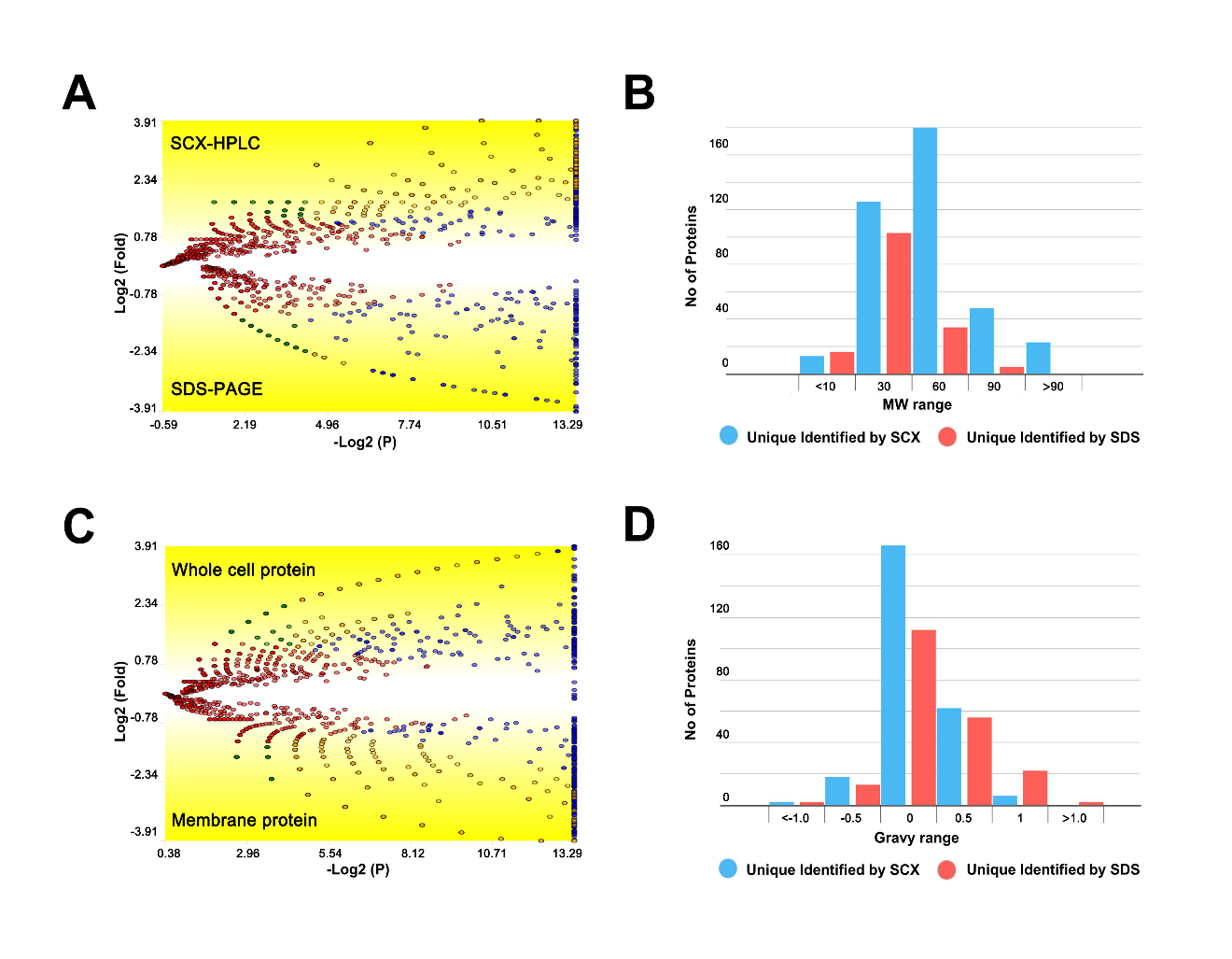


**Figure S2 The unique proteins identified by different proteomics strategies in this study.** (A) In-gel fractionation (SDS-PAGE) and in-solution fractionation (SCX) differential according to the Patternlab ACFold principal component analysis. The plot on the right shows the distribution of the identified proteins according to the ordinate (y) and *P*-value on the abscissa (x). The plot tab indicates that proteins (blue dots) were differentially expressed between in-gel fractionation and in-solution fractionation. (B) Differences in the MW range of unique proteins identified by SCX and SDS-PAGE. (C) Whole cell protein fractionation and membrane protein fractionation differential according to the Patternlab ACFold principal component analysis. The plot tab indicates that proteins (blue dots) were differentially expressed between whole cell protein fractionation and membrane protein fractionation. (D) Differences in the GRAVY score range of the unique proteins identified by whole cell protein fractionation and membrane protein fractionation.


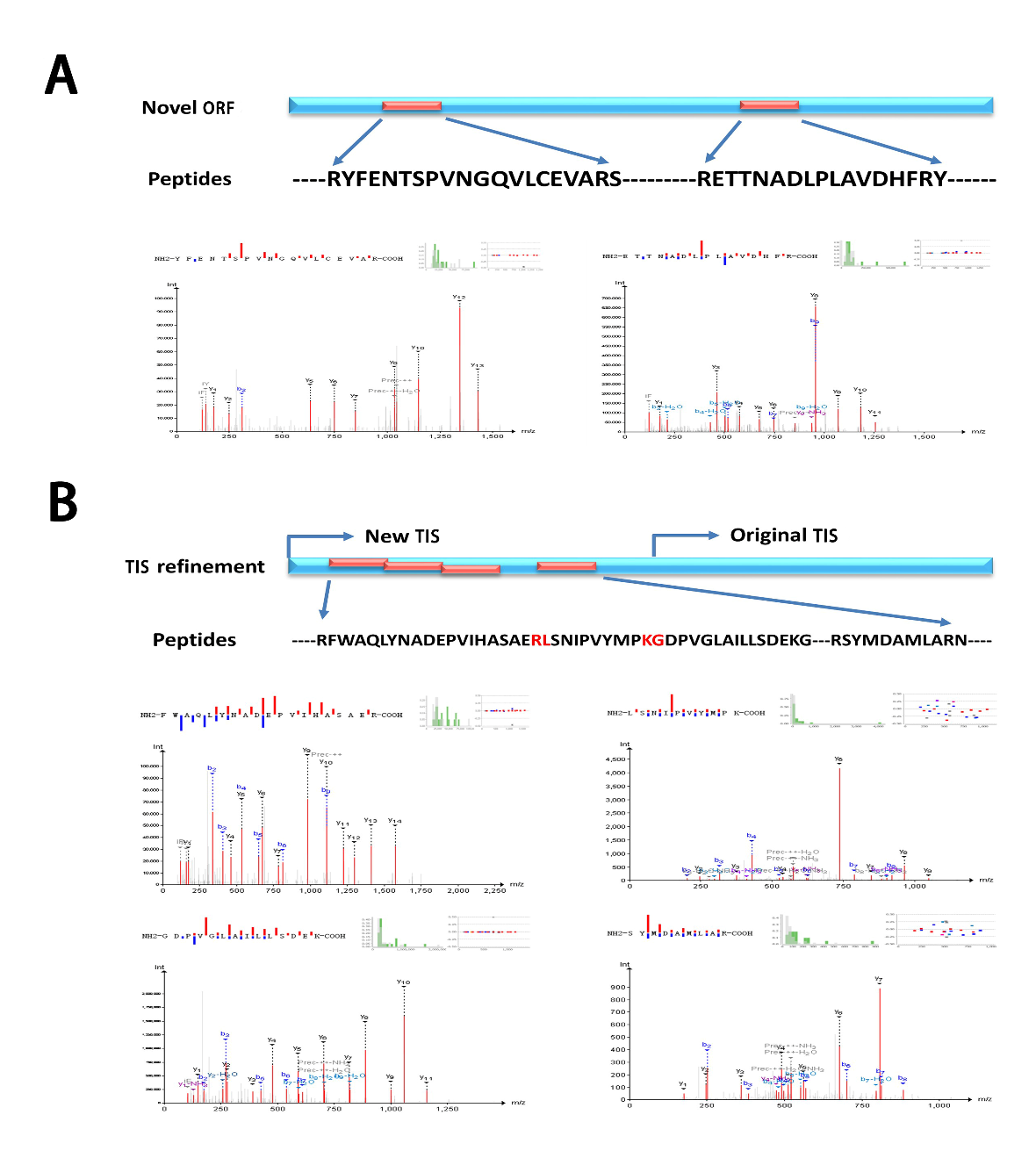


**Figure S3 Refinement of genome annotation by proteogenomic analysis in this study.** (A) Two unique peptides (RYFENTSPVNGQVLCEVARS, RETTNADLPLAVDHFRY) were mapped to a novel ORF (NZ_CP009625_3701). (B) Four peptides (RFWAQLYNADEPVIHASAER, RLSNIPVYMPK, GDPVGLAILLSDEKG, RSYMDAMLARN) were mapped to match the upstream region of an existing ORF (NZ_CP009626_26949). Blue colored peaks are matched b-ion series. Red colored peaks are matched y-ion series. Black colored peaks are unassigned experimental peaks
